# Histopathology and Spatial Distribution of Putative Growth Factors in Relation to Bacterial Localization of *Campylobacter jejuni* Within the Ovine Gallbladder

**DOI:** 10.3389/fvets.2019.00226

**Published:** 2019-07-12

**Authors:** Amanda J. Kreuder, Victoria Lashley, Michael Yaeger, Jennifer A. Schleining, Paul J. Plummer

**Affiliations:** ^1^Department of Veterinary Diagnostic and Production Animal Medicine, College of Veterinary Medicine, Iowa State University, Ames, IA, United States; ^2^Department of Veterinary Microbiology and Preventive Medicine, College of Veterinary Medicine, Iowa State University, Ames, IA, United States; ^3^Department of Veterinary Pathology, College of Veterinary Medicine, Iowa State University, Ames, IA, United States

**Keywords:** *Campylobacter*, ovine, gallbladder, bile, growth factors

## Abstract

*Campylobacter jejuni* is an important zoonotic pathogen that is the leading cause of both human foodborne bacterial gastroenteritis worldwide and ovine abortion in the United States. Previous studies have demonstrated that the gallbladder of ruminants is often positive on culture for *Campylobacter* sp., suggesting that this environment may serve as a chronic nidus of infection for maintenance of disease within populations. The objective of this study was to determine if previously identified putative growth promoting factors of *C. jejuni* are present within the gallbladder mucosa of sheep and to evaluate for bacterial co-localization of *C. jejuni* with these compounds following experimental inoculation. Direct gallbladder inoculation with *C. jejuni* sheep abortion (SA) clone clinical isolate IA3902 followed by immunohistochemical analysis and scanning electron microscopy allowed for identification of *C. jejuni* at the gallbladder mucosal surface and within the gallbladder submucosal glands. Histochemistry identified several putative *Campylobacter* growth promoting factors including neutral and acid mucins as well as L-fucose to be present both on the mucosal surface as well as in the gallbladder submucosal glands. In summary, following experimental inoculation of the ovine gallbladder, *C. jejuni* IA3902 was identified in direct contact with the gallbladder mucosal surface and deep mucosal glands in the same location as several putative growth promoting factors. This suggests the yet to be tested hypothesis that under natural conditions of infection, the gallbladder submucosal glands have the potential to provide a protected niche for chronic carriage of *C. jejuni* in animal hosts.

## Introduction

Campylobacteriosis is an important infectious disease of sheep worldwide, leading to abortion storms with large economic losses in infected flocks. Historically, *C. fetus* subsp. *fetus* was considered the primary causative agent of ovine abortion in the United States due to *Campylobacter* sp., with only sporadic cases of abortion due to *C. jejuni* of varying strain types reported (1, 2). Since the late 1980's, however, there has been a steady increase in the percentage of ovine abortions attributed to *C. jejuni* in the U.S., with isolates of *C. jejuni* outnumbering *C. fetus* subsp. *fetus* by the mid 1990's (1, 3). Between the end of the last century and the 2000's, a single clonal isolate, *C. jejuni* sheep abortion (SA) clone, represented by the clinical isolate IA3902, was observed to become the predominant cause of ovine abortion in the United States (4). Outbreaks of zoonotic transmission of this hypervirulent strain to humans, primarily related to raw milk consumption, have been increasingly reported over the last decade (5), highlighting the need for greater understanding of the mechanisms utilized by this virulent strain of *C. jejuni* to both cause disease and persist in animal hosts.

Similar to the ecology of the organism in chickens, it is suspected that chronic colonization of the ruminant host leads to constant shedding of the *C. jejuni* into the environment thus maintaining it in the sheep population even when clinical disease is not present. The majority of the work done to detect chronic carriage of *C. jejuni* in other reservoir species such as chickens has been performed looking at chronic colonization of the intestinal environment [reviewed in (6)]; however, a positive culture from feces or intestinal contents does not necessarily prove that the intestines themselves are the home for chronic colonization. Constant bile secretion from the gallbladder into the intestinal tract provides an alternative location for chronic *C. jejuni* carriage that could lead to a positive fecal culture result.

Multiple studies performed on abattoir samples of bile from sheep and other ruminants have demonstrated that *C. jejuni* can frequently be isolated from the gallbladders of otherwise healthy animals (5, 7, 8). While *Campylobacter* sp. can frequently be found within the gallbladder, it is unclear how the bacteria reach the gallbladder as both the common bile duct and via portal circulation are both options. It is also unclear whether they survive primarily as free-living bacteria in the bile or by colonizing the protective mucous layer as has been demonstrated to play an important role in intestinal colonization by *Campylobacter* in other animal species (9). To decrease both the burden of disease in animal populations as well as decrease the risk of zoonotic transmission to humans, there is a critical need to understand how *C. jejuni* is able to not only colonize but potentially thrive in this otherwise harsh environment. Previous studies have shown that mucins, L-fucose, and iron all can serve as chemoattractants for *C. jejuni* in other locations within the ovine host, such as the placenta (10). Thus, we hypothesized that the ovine gallbladder mucosa and mucosal glands may produce chemoattractant compounds with growth promoting characteristics that may make the gallbladder mucosal surface an attractive location for *C. jejuni* colonization.

To test this hypothesis, a previously developed unique *in vivo* model for gallbladder inoculation in sheep was utilized to provide a rapid assessment of the localization of *C. jejuni* within the gallbladder following exposure. In addition, we utilized previously described histochemical methods to confirm that many of the compounds previously identified to be chemoattractive to *C. jejuni* are present within the mucosal lining and deep glands of the ovine gallbladder. Under these experimental conditions, our findings suggest that chemoattractant compounds present in the deep mucosal invaginations and glands of the gallbladder mucosa may encourage recruitment of *C. jejuni* into these crypts. Further studies are warranted to determine if this location may provide a protected niche where *Campylobacter* can survive and replicate to establish a chronic nidus of infection under conditions of natural infection within the ruminant host.

## Materials and Methods

### Bacterial Strains and Preparation of Animal Inoculum

A clinical isolate of *C. jejuni* SA clone (IA3902), which has been frequently identified from the gallbladder of sheep in abattoir studies (5), was utilized for this study. *C. jejuni* IA3902 was routinely grown in Mueller-Hinton (MH) broth or agar plates (Becton-Dickinson, Franklin Lakes, NJ) at 42°C under microaerophilic conditions with the use of compressed gas (55% O_2_, 10% CO_2_, 85% N_2_). Preparation of the animal inoculum was identical to the methods described previously in Kreuder et al. (11).

### Inoculation of the Sheep Gallbladder With *C. jejuni* IA3902

All animal experiments were approved by the Iowa State University Institutional Animal Care and Use Committee (IACUC) prior to initiation and followed all appropriate animal care guidelines. Experimental inoculation of *C. jejuni* IA3902 into the gallbladder of 8 adult female mixed breed sheep was performed via full laparotomy with placement of a Hemoclip® over the common bile duct as previously described (11) with euthanasia performed at either 2 or 24 h post-inoculation. Immediately following humane euthanasia, the abdomen was entered and bile was removed sterilely via gentle aspiration with a 16 gauge 1” needle and a 60 mL syringe.

Following removal of the bile contents, the gallbladder was then removed in its entirety and further samples collected for histopathology, immunohistochemistry, and electron microscopy as described below. Following collection of these samples, approximately half of the gallbladder wall remained; this tissue was then rinsed with sterile saline to remove loose droplets of bile and then the entire mucosal surface was scraped with a straight edge sterile blade to remove the mucous layer and mucosal lining. This material was then collected into sterile 15 mL conical tubes and the volume in the vials was standardized to 3 mL each using sterile saline. The collected material was vortexed vigorously for 1 min and 100 μl of this solution was used for a serial dilution in MH broth to provide a semi-quantitative estimate of the amount of *C. jejuni* contained within the mucus layer of the gallbladder. The amount estimated in CFU/mL was then multiplied by 3 mL based on the starting volume of the mucosal scraping solution; this value was again multiplied by 2 based on the fact that only half of the gallbladder wall was utilized for this purpose to determine an estimate of total bacterial numbers present within the gallbladder mucosa.

Gallbladder samples were collected at necropsy of an additional group of eight sheep obtained from one of the same farms as above that were being utilized as controls for an unrelated study. The gallbladder of these animals was removed in its entirety and fixed in 10% neutral buffered formalin for histology and immunohistochemistry as non-inoculated controls; only samples confirmed to be free of culturable bacteria including *Campylobacter* were used for this purpose. Confirmation of culture negative status of the bile was performed as described previously and results reported in Kreuder et al. (11).

### Bacterial Culture From Bile and Gallbladder Mucosa

Prior to inoculation, 1 mL of bile was removed from each gallbladder and cultured as previously described (11) to determine if culturable bacteria were present prior to inoculation. Of the 8 animals, 6 of the 8 were culture negative prior to inoculation. One sheep did not have any bile present in the gallbladder for culture; this animal was screened via fecal culture and found to be negative for *Campylobacter* sp. The final animal did display pure growth (<10^5^ CFU/mL) of colonies that were identified to be *C. jejuni* via MALDI-TOF mass spectrometry (Bruker Daltonics, Billerica, MA); samples from this animal were not utilized for additional data collection beyond bacterial culture and routine hematoxylin and eosin staining. Immediately following sample collection and processing, 100 μL from each bile sample [average result previously reported in Kreuder et al. (11) in comparison to average results of IA3902 inocultated into sheep bile *in vitro*] as well as 100 μl of mucosal scraping were used to determine the viable CFU/mL of IA3902 in the inoculated sheep gallbladder. Serial dilution onto MH plates and incubation at 42°C microaerophilic using the drop-plate method was performed as previously described (12). Both individual and average culture results for both bile and gallbladder scraping samples were determined, and statistical analysis of differences in the amount of bacteria cultured between the bile and mucosal scrapings as well as between 2 and 24 h of incubation was performed via a two-way ANOVA (not repeated measures) with Sidak's multiple comparisons test (GraphPad, Prism).

### Gallbladder Histology and Histochemistry

Following removal of bile from the gallbladders, samples of the gallbladder wall were placed in 10% neutral buffered formalin and submitted to the Iowa State University College of Veterinary Medicine Comparative Pathology Core Service for histology, histochemistry, and immunohistochemistry processing and evaluation.

To prepare the samples for evaluation, serial sections of each formalin fixed sample of gallbladder wall were embedded in paraffin and sections cut to 5 μm thickness. Cut sections were then stained with hematoxylin and eosin (H & E) for routine histological examination as well as Perl's iron stain, Alcian blue (pH 2.5), and the periodic acid-Schiff reaction with and without diastase pre-treatment to identify the presence or absence of material with staining characteristics consistent with iron, acid mucins, and neutral mucins, respectively.

Lectin histochemistry to identify L-fucose containing glycans was performed as previously described utilizing the commercially available lectins biotinylated *Ulex europaeus* agglutinin I (UEA-I; Vector Laboratories, Burlingame, CA) and biotinylated *Lotus tetragonolobus* lectin (LTA; Vector Laboratories) and visualized using a commercial kit (Vectastain Elite ABC; Vector Laboratories) and chromogen (NovaRED; Vector Laboratories) (10).

### Gallbladder Immunohistochemistry

To determine the location of *C. jejuni* bacteria within the sections of gallbladder, immunohistochemistry directed against the major outer membrane protein (MOMP) of *C. jejuni* was performed on a subset of randomly chosen gallbladder samples (two each from the 2 h and 24 h inoculated sheep, and one uninoculated control) as previously described (13). The primary antibody, which was directed against the major outer membrane protein of *C. jejuni*, was prepared as previously described (14) and used at a dilution of 1:300; a commercially available biotinylated secondary antibody (MultiLink; Biogenex) was used at a dilution of 1:80.

### Scanning Electron Microscopy of Gallbladder Mucosa

Additional sections of gallbladder wall were collected separately at necropsy and fixed in 2% paraformaldehyde and 3% glutaraldehyde in 0.1 M cacodylate buffer at 4°C for 24 h then submitted to the Iowa State University Microscopy and NanoImaging Facility to be prepared for scanning electron microscopy (SEM). A single sample representative of each time point (2 h and 24 h) was selected for further processing based on evidence of normal mucosal architecture present in the histopathology examination. Fixed samples were rinsed in deionized water and post-fixed in 2% aqueous osmium tetroxide followed by dehydration in a graded ethanol series up to 100% ultra-pure ethanol and dried using a Denton DCP-2 critical point dryer (Denton Vacuum, Moorestown, NJ). When dried, the samples were placed onto adhesive coated aluminum stubs, sputter coated (Denton Desk II sputter coater, Denton Vacuum) with palladium/gold alloy, and imaged using a JEOL 5800 LV scanning electron microscope (Japan Electron Optics Laboratory, Peabody, MA) at 10 kV.

## Results

### Culture of Bile and Gallbladder Mucosa

**Figure 1:**
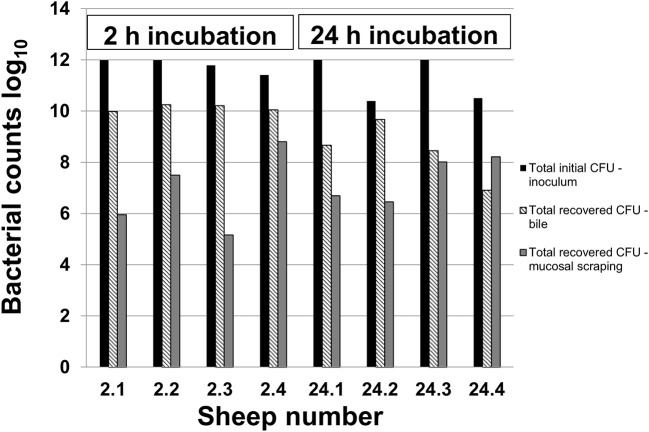
Recovery of *C. jejuni* in bile and mucosal scrapings following inoculation. Samples of bile and mucosal scrapings following 2 and 24 h incubation of *C. jejuni* inoculated directly into the ovine gallbladder were cultured to estimate total remaining CFU using serial dilutions and the drop plate method of enumeration.

Figure 1 demonstrates the inoculated amount of *C. jejuni* IA3902 compared with the amount recovered from both the bile and mucosal scrapings of each individual animal. The average inoculum was 11.5 log10 (6 × 10^11^ total CFU). After 2 h of incubation, an average of 10.1 log10 bacteria were collected out of the bile, with an additional 6.9 log10 bacteria present in the gallbladder wall scrapings. Following 24 h of incubation, an average of 8.4 log10 bacteria were present in the bile, with an additional 7.3 log10 bacteria estimated to be located within the gallbladder wall scrapings. Statistical analysis via two-way ANOVA did not demonstrate a statistically significant difference between these two locations and time points.

### Routine Histology Findings, Immunohistochemistry and SEM

**Figure 2:**
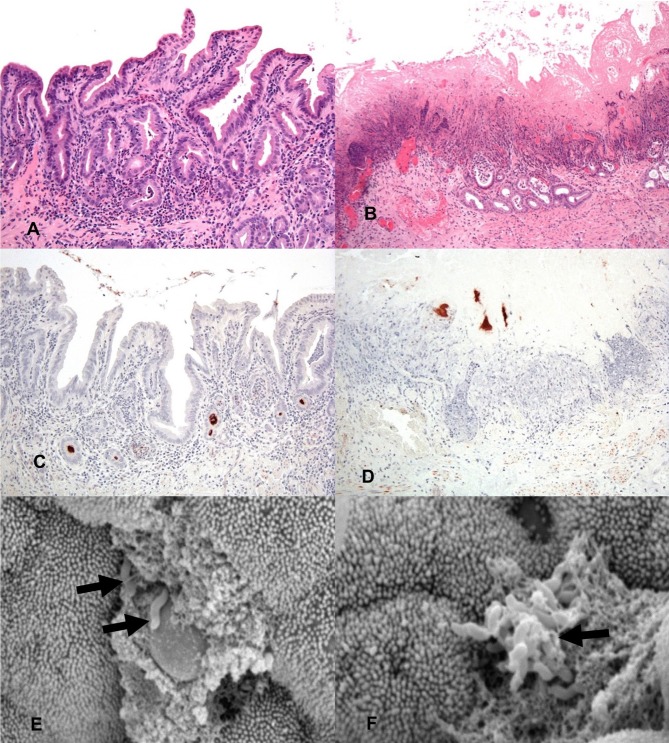
General histology (HE) of inoculated gallbladders and localization of *C. jejuni* within the gallbladder via IHC and SEM. **(A)** 2 h post-inoculation there are mild to moderate diffuse inflammatory infiltrates in the lamina propria mildly separating and surrounding mucosal invaginations. There is distension of the basal aspect of multifocal mucosal invaginations by inflammatory cells and cellular debris. Hematoxylin and eosin (HE). **(B)** 24 h post-inoculation the mucosal architecture is diffusely disrupted, the apical mucosa is necrotic, moderate numbers of glands are lined by attenuated epithelium, are ectatic and partially filled with mucus, degenerate neutrophils and pkynotic cellular debris. Hematoxylin and eosin (HE). **(C)** IHC staining for *C. jejuni* (brown) shows aggregates of organism deep within mucosal invaginations, on the mucosal surface, and in luminal debris 2 h post-inoculation. **(D)** IHC staining for *C. jejuni* (brown) shows organisms present in the necrotic remnants of mucosal invaginations 24 h post-inoculation. **(E)** A focal ulceration, with two intralesional *C. jejuni* organisms (arrows). Scanning electron micrograph (SEM). **(F)** Scanning electron micrograph (SEM) of the gallbladder mucosal surface with an aggregate of *C. jejuni* organisms (arrow) adherent to the apical aspect of the mucosa with associated microvillus loss, clumping and blunting of adjacent microvilli.

A summary of the routine histology findings of the sheep gallbladder samples is presented in Table 1 with representative images of 2 h and 24 h samples presented in Figures 2A,B.

**Table 1:** Summary of histologic changes associated with direct inoculation of the ovine gallbladder with *C. jejuni*.

		**Mucosal changes**						**Muscularis changes**		**Serosal changes**
	**Sheep ID**	**Ulceration**	**Gland abscesses**	**Eosinophils within the mucosa and lamina propria**	**Mott cells within the mucosa and lamina propria**	**Lymphoid nodules**	**Neutrophilic infiltration**	**Vascular congestion/lymphatic dilation**	**Edema**	**Neutrophilic infiltration**
2 h	2.1^a^	0	3	2	1	1	2	1	3	1
	2.2^b^	0	1	2	1	0	2	1	2	2
	2.3	0	3	0	3	1	1	3	2	0
	2.4^b^, ^c^	0	1	2	2	2	2	1	1	0
24 h	24.1^b^	1	2	1	0	1	3	3	2	3
	24.2^b^	3	0	2	3	0	3	3	3	1
	24.3	Unable to score due to 2-3 cm of transmural necrosis, demarcated by a band of degenerate neutrophils and cellular debris								
	24.4^c^	0	2	1	1	2	2	1	1	2
Control^d^	1	0	0	0	1	2	0	1	0	0
	2^b^	0	0	0	1	1	0	1	0	0

*0 = none/no lesion of that type present; 1 = mild lesion present; 2 = moderate lesion present; 3 = severe lesion present*.

a *pre-inoculation bile positive for growth of C. jejuni*.

b *sample processed for immunohistochemistry*.

c *sample processed for SEM*.

d *gallbladder samples taken from uninoculated sheep*.

To determine the location of *C. jejuni* within the gallbladder environment, immunohistochemistry directed toward the major outer membrane protein (MOMP) of *C. jejuni* was performed on a subset of the sheep gallbladder samples. Immunohistochemistry of the selected samples identified dense accumulation of organisms deep within mucosal invaginations and glands of the gallbladder and as multifocal aggregates within clumps of luminal debris and mucus. By 2 h after inoculation, both samples examined were observed to have *C. jejuni* located deep within many of the mucosal invaginations and glands (Figure 2C). By 24 h, as much of the mucosal surface exhibited severe inflammation and necrosis, the majority of the *C. jejuni* was observed to be present in aggregates adhered to mucin and inflammatory debris on the surface of the mucosa, however, where still visible, *C. jejuni* could be observed to be located within mucosal invaginations and submucosal glands (Figure 2D). For all slides of inoculated gallbladders examined, there was also staining observed at the serosal surface indicative of leakage of inoculated bile from the puncture site. In contrast, no staining indicative of *C. jejuni* presence was noted either on the mucosal or serosal surface in the culture negative uninoculated gallbladder samples.

In addition to immunohistochemistry, scanning electron microscopy of a subset of the inoculated gallbladder samples (one each from the 2 h and 24 h time points) was also utilized in an attempt to locate *C. jejuni* on the surface of the gallbladder mucosa. Figure 2E demonstrates an aggregate of *C. jejuni* organisms at 2 h post-inoculation closely adherent to the apical aspect of the mucosal surface located near a small focus of microvilli loss. The adjacent material within which the *C. jejuni* is located is likely made up of cellular debris and mucus. Figure 2F, also taken at 2 h post-inoculation, demonstrates two *C. jejuni* organisms adherent to a focally extensive area of ulceration, again with adjacent material that is likely to be made up of cellular debris and mucus. These images demonstrate that within a relatively short amount of time post-inoculation, *C. jejuni* is able to migrate through the mucous layer to become intimately associated with the gallbladder mucosa. Additional organisms were observed within the spaces between villi; however, the microscope could not be focused to those regions to capture images.

### Histochemistry

To determine if any factors previously known to be tropic for *C. jejuni* were present in the same locations in the ovine gallbladder as *C. jejuni* identified via immunohistochemistry, an additional subset of slides were stained with Perl's iron stain, Alcian blue (pH 2.5), lectin staining, and the periodic acid-Schiff (PAS) reaction with and without diastase pre-treatment. Perl's iron stain did not reveal appreciable staining within mucosa, submucosa, muscularis, or serosa of sections examined, indicating that iron accumulation is not a feature of the ovine gallbladder wall.

**Figure 3:**
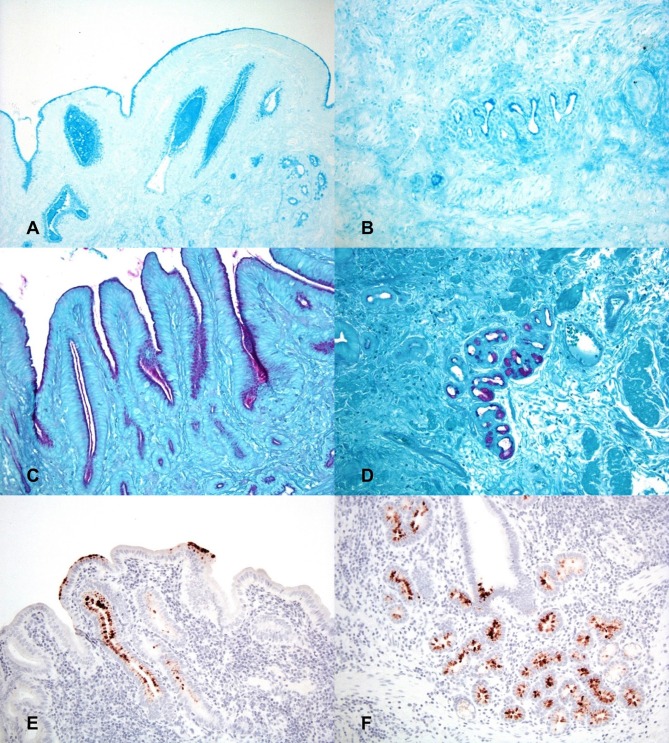
Histochemical staining for putative growth factors in gallbladders inoculated with *C. jejuni*. **(A)** The mucosal surface and deep invaginations are lined with moderate amounts of acid mucin (deep blue staining) and there are pools of acid mucin within the lumen of invaginations at 2 h post-inoculation. Alcian blue (pH 2.5). **(B)** At 24 h post-inoculation acid mucin staining is largely absent in the necrotic surface mucosa but is still marked within the cells and the lumen of deep mucosal invaginations. Alcian blue (pH 2.5). **(C)** The apical cytoplasm of mucosal epithelial cells and the lumen of mucosal invaginations contain large amounts of neutral mucin (purple staining) 2 h post-inoculation. PAS staining. **(D)** Neutral mucin staining is also marked within submucosal glands. PAS staining. **(E)** 2 h post-inoculation the mucosal surface and mucosal invaginations are lined with large amounts of L-fucose (brown staining). Lectin staining for L-fucose. **(F)** The deep mucosal invaginations and submucosal glands also contain large amounts of L-fucose 2 h post-inoculation. Lectin staining for L-fucose.

Alcian blue staining was utilized to identify the presence of acid mucins within sections of gallbladder at both 2 h and 24 h. Both time points demonstrated marked stain uptake within the cells and lumen of the deep crypts, extending throughout the gland lumen space and multifocally dispersed among gallbladder debris (Figures 3A,B). Staining via PAS was utilized to identify the presence of neutral mucins within sections of gallbladder at both 2 h and 24 h. Again, both timepoints displayed marked PAS staining multifocally at the deep aspect of glands and as aggregates and streaming bands within the gallbladder lumen (Figures 3C,D).

Lectin staining was also utilized to determine if L-fucose containing glycans were present within the gallbladder mucosa. Both time points displayed multifocal areas of variable to strong lectin staining within glands and on the surface of the epithelium (Figures 3E,F). Based on the staining patterns for both acid and neutral mucins, as well as L-fucose, the observed areas of MOMP immunohistochemistry correspond well to the same locations of putative *C. jejuni* growth factors, particularly deep within the mucosal invaginations and glands of the gallbladder.

## Discussion

The mammalian gallbladder environment is typically thought of as “harsh” with few bacteria able to survive under the inhospitable conditions. Bile acids (salts) are the main component of bile, along with cholesterol, phospholipids, and bilirubin. Their amphipathic nature allows them to act as a detergent which plays a key role in lipid solubilzation and emulsification leading to digestion of fats within the intestinal tract (15). Studies have demonstrated that sheep bile, along with ox and pig bile, has a relatively high percentage of bile salts which constitute 10% w/v of the total contents of bile. The presence of high concentrations of bile salts have been proven to be very damaging to cellular membranes (16), and the detergent properties of bile has been demonstrated to have potent antimicrobial activity (17). For evidence of the inhospitable nature of the gallbladder environment, one need not look farther than the exit of the common bile duct into the duodenum; while the gallbladder often remains free of culturable bacteria, the adjacent intestine separated by only a short distance is home to a wide array of culturable bacterial species.

However, the relatively frequent isolation of *C. jejuni* from bile samples of multiple ruminant species, typically in the absence of other culturable bacteria, suggests that the gallbladder may in fact serve as a protected niche for chronic colonization by bacteria adapted to survive within its walls (5, 7, 8). This phenomenon may not be limited to ruminants as multiple instances of cholecystitis in humans due to *Campylobacter* sp. have been reported (18, 19). Other intestinal pathogens such as *Salmonella typhi* and *Listeria monocytogenes* have also been shown to have the unique ability to colonize the gallbladder in both humans and animals where they can establish a chronic carrier state in their host (20, 21). In addition, the DNA of the closely related *H. pylori* has also been demonstrated to be frequently present in the gallbladder of patients with cholelithiasis although direct culture has proven more challenging (22).

The regular flushing action of frequent bile secretion, particularly in species like sheep where biliary secretion is tied to rumination which occurs throughout the day, should make chronic colonization of the gallbladder difficult for organisms that exist exclusively as free-living within the gallbladder lumen. Previously published data suggests that sheep secrete up to 40 μl of bile/kg/minute which is seven times higher than the endogenous rate of bile flow in monogastrics such as dogs (23). Therefore, it is reasonable to suggest that long-term survival by *C. jejuni* within the ovine gallbladder requires a mechanism to counteract the constant flushing action of bile release.

Intestinal colonization with *C. jejuni* is associated with the ability to colonize the mucin and L-fucose-containing mucous layer of the intestinal epithelium where it is protected from the mechanical and chemical milieu of the intestinal lumen (24, 25). Unlike many other enteric pathogens that become trapped in this layer, *C. jejuni* is able to move freely within the mucin layer to inhabit the deep intestinal crypts (26) and from there potentially become internalized within eukaryotic cells (27–29). This ability has also been demonstrated in the closely related *H. pylori* to allow colonization of the glands of the stomach (30). Mucin, L-fucose, and bile have all been shown to be strong chemoattractants for *C. jejuni* (31) and it has been demonstrated that some virulent strains of *C. jejuni* such as IA3902 can utilize L-fucose as a substrate for growth due to possession of a specific genetic island encoding a region for fucose metabolism (32, 33).

The studies of the growth and chemotaxis of *Campylobacter* spp described above, as well as additional studies evaluating tropism of *C. jejuni* IA3902 within the guinea pig placenta (10), provided potential target compounds to evaluate within the sheep gallbladder including mucins, L-fucose, and iron. The data presented in our work clearly demonstrates that many of the same factors thought to be chemotropic to *C. jejuni* in the guinea pig placenta, such as neutral and acid mucins and L-fucose, are also present in the ovine gallbladder. In addition, the presence of MOMP staining indicative of *C. jejuni* antigens within the same locations as the chemoattractive mucins and L-fucose strongly suggests that *C. jejuni* has an affinity for these locations due to their enhanced presence in the same regions. The location of *C. jejuni*, as detected by immunohistochemistry, within the deeper aspects of the mucosal invaginations and glands of the gallbladder mucosa was consistent, even in severely inflamed gallbladder mucosa. The large aggregates of *C. jejuni* organisms present within the deep aspects of the glands also suggest that replication of organisms may be occurring in those locations. Thus, is seems reasonable to hypothesize that these protected areas of the gallbladder may provide a protected location and thus a defense against the harsh luminal environment and constant flushing action of bile release. If colonization occurs in this location, it would potentially allow bacterial resources to be dedicated to replication rather than purely survival. Scanning electron microscopy also allowed an unprecedented view of *Campylobacter* organisms in direct contact with the microvilli on the surface of the gallbladder epithelium; their location in association with what appears to be extracellular debris is also suggestive of an affinity for mucins. The culture data presented in this study is supportive but not conclusive of migration of *C. jejuni* from free-living organisms within the gallbladder lumen toward the more protected niche of the mucous layer and glands of the gallbladder wall.

Factors that may lead to tropism of *C. jejuni* to the gallbladder have not been described in any species. The effect of bile itself as a chemoattractant for *Campylobacter* has been variable depending on the species studied. Interestingly, while *C. jejuni* can be cultured out of the guinea pig gallbladder, the bile from guinea pigs has been shown to be chemorepellent (13). In contrast, diluted bovine and chicken bile have been described as chemoattractive for certain *C. jejuni* strains isolated from chickens; when the mucin fraction of the bile was removed, however, the remaining bile salts were universally chemorepellent (31). This suggests that biliary mucins may act as the chemoattractive fraction in bile. Our data which demonstrated aggregates of mucin staining within the lumen and deep glands of the gallbladder in conjunction with MOMP staining of *C. jejuni* within the same regions are supportive of the theory that mucins in bile may play a role in chemoattraction to the ovine gallbladder. The composition of bile salts varies greatly between species and may also play a role in observed differences in chemoattraction between bile from different species (34). It is possible, therefore, that the composition of the bile salts, may also play a role in the affinity of *C. jejuni* to the gallbladder environment based on the host species.

In summary, the data presented herein has demonstrated that previously identified putative growth factors for *Campylobacter jejuni* including neutral mucin, acid mucin, and L-fucose are present in the ovine gallbladder. These areas include the deeper aspects of the mucosal invaginations and mucosal glands of the gallbladder and in free-floating luminal debris composed of neutral and acid mucin aggregates. Under experimental inoculation conditions, aggregates of *C. jejuni* were also noted to be present in the same locations. This data is supportive of the hypothesis that to survive with the harsh environment of the gallbladder, colonization of the deep mucosal glands may occur to allow avoidance of the constant flushing action of bile release and the detergent activities of bile salts in the lumen. Future work in models utilizing natural methods of *C. jejuni* experimental inoculation or naturally infected animals is warranted to confirm the observed experimental location of *C. jejuni* within the gallbladder environment as well as determine the route by which gallbladder colonization occurs.

## Data Availability

All datasets generated for this study are included in the manuscript and/or the supplementary files.

## Ethics Statement

This study was carried out in accordance with the recommendations of and approved by the Iowa State University Institutional Animal Care and Use Committee (IACUC) prior to initiation and followed all appropriate animal care guidelines.

## Author Contributions

AK was responsible for the design and execution of all of laboratory work, animal studies, and manuscript preparation. PP and JS assisted with the study design and completion of animal studies, and editing the manuscript. MY was responsible for necropsy and sample collection from the sheep gallbladder. MY and VL were responsible for completion of the histology, histochemistry, and immunohistochemistry. All authors reviewed the manuscript and approved of the final version.

### Conflict of Interest Statement

The authors declare that the research was conducted in the absence of any commercial or financial relationships that could be construed as a potential conflict of interest.
